# Innovating Household Food Waste Management: A User-Centric Approach with AHP–TRIZ Integration

**DOI:** 10.3390/s24030820

**Published:** 2024-01-26

**Authors:** Shuyun Wang, Hyunyim Park, Jifeng Xu

**Affiliations:** 1 School of Design, The Hong Kong Polytechnic University, Hong Kong, China; 2 School of Art, Southeast University, Nanjing 211189, China

**Keywords:** food waste management, smart system design, user-centric design, AHP–TRIZ integration, household waste reduction

## Abstract

Food waste management remains a paramount issue in the field of social innovation. While government-led public recycling measures are important, the untapped role of residents in food waste management at the household level also demands attention. This study aims to propose the design of a smart system that leverages sensors, mobile terminals, and cloud data services to facilitate food waste reduction. Unlike conventional solutions that rely on mechanical and biological technologies, the proposed system adopts a user-centric approach. By integrating the analytical hierarchy process and the theory of inventive problem solving, this study delves into users’ actual needs and explores intelligent solutions that are alternatives to traditional approaches to address conflicts in the problem solving phase. The study identifies five main criteria for user demands and highlights user-preferred subcriteria. It determines two physical conflicts and two technical conflicts and explores corresponding information and communications technology (ICT)-related solutions. The tangible outcomes encompass a semi-automated recycling product, a mobile application, and a data centre, which are all designed to help residents navigate the challenges regarding food waste resource utilisation. This study provides an approach that considers users’ genuine demands, empowering them to actively engage in and become practitioners of household food waste reduction. The findings serve as valuable references for similar smart home management systems, providing insights to guide future developments.

## 1. Introduction

China’s urbanisation and economic development continue, especially with the expansion of the restaurant and food delivery industries in recent years. However, along with this trend, the issue of food waste generation in Chinese urban areas has become increasingly serious. Estimates showed that, in 2020, China’s food waste amounted to 61.37 Mt, reflecting a significant increase of 29.67% in per capita food wastage compared to the 2016 figures, and this trend is anticipated to escalate in the future [1]. Food waste contains a large amount of organic matter. On the one hand, it has posed a potential threat of pollution to water and land resources, as well as the spread of germs that affect people’s physical health. On the other hand, it has great value for resource reclamation as it can be collected and converted into value-added products [2]. Hence, investigating strategies for the collection, management and efficient conversion of food waste has emerged as a major research topic in recent years.

China is making great efforts to achieve its *zero-waste city* goal, which refers to an urban development model that aims at recycling solid waste and reducing landfills by promoting green development and green lifestyles. Garbage classification is one of the most common measures. Food waste is sorted and utilised to produce biomass fuel and natural gas [3]. Although these actions have been implemented in some pilot cities for several years, their effectiveness remains low. As a comprehensive project, waste recycling in China is currently managed through an intensive government-led approach [4], which requires overall process management, including policy measures, source-separated collection, transportation, treatment and disposal [5]. Consequently, problems such as mixed collection and low efficiency in resource utilisation are common wicked issues that affect this endeavour [6]. In addition to the government-led mode, an alternative approach worth exploring is a user-centric perspective, which is a decentralised and more direct household-focused solution to food waste management.

Many studies have proposed strategies for improving residents’ awareness of effective recycling [4,7], but there is a lack of specific solutions on how to facilitate and support residents’ recycling behaviour. Most Chinese households are not equipped with practical products to process food waste. Therefore, improving the household resource recycling management system to match residents’ environmental awareness is important as it helps to achieve an effective combination of awareness and appropriate recycling measures [8]. Seeking suitable disposal approaches for household food waste management is a necessary and meaningful topic.

**Figure 1:**
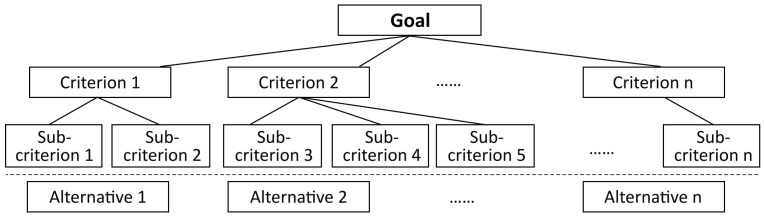
The AHP model.

**Figure 2:**
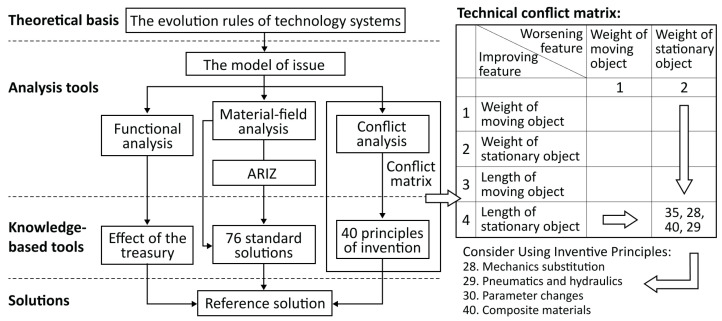
Flowchart of the TRIZ theory and an example of the conflict matrix.

To provide a practical household disposal solution and improve residents’ recycling experience, this study proposes a user-centric approach for managing household food waste in a smarter way based on the analytical hierarchy process (AHP) and the theory of inventive problem solving (TRIZ). The AHP is a structured multi-attribute decision making method (see Figure 1) in which the criteria of an issue are organised in a hierarchical structure and relatively compared to determine their order of priority [9]. The theory of inventive problem solving consists of tool sets generated from massive inventions to address the challenges within a technical system, thus improving its functionality (see Figure 2) [10]. Based on the proposed approach, modern technological components, such as wireless sensors and mobile terminals, can be integrated into the final system design solution. These technologies enable real-time data collection, enhance user engagement and streamline the management of household food waste [11]. By combining user research methods with information and communications technology (ICT), a holistic and efficient solution can be developed to effectively manage food waste while fostering residents’ environmental awareness.

The main contributions of this study are as follows:

- Explore factors and identify user preferences regarding the household food waste recycling issue.
- Present a theoretical approach for designing the household food waste management system, which can be applied to other household smart management issues and has the potential to evolve conventional products into smart management systems.
- Develop a practical solution incorporating smart technology to manage household food waste recycling based on the proposed approach.

The remaining sections are structured as follows. Section 2 discusses the background of food waste recycling in Chinese communities and similar initiatives in terms of products, biotechnology and smart systems. Section 3 contextualises the research method’s architecture and discusses the approach step by step. Section 4 presents the design generated from the previous analysis. Finally, Section 5 discusses the findings from the proposed approach in the field of smart home system design.

## 2. Related Work

As China is experiencing a growing amount of food waste every year, the implementation of the wet waste classification policy in the country has accelerated. In 2019, 16 cities in China initiated the pilot construction of *zero waste cities*, a move aimed at promoting source reduction and resource utilisation of solid waste [12]. The most widely adopted approach involves collecting a large proportion of waste from the community or street scale, which then undergoes processing through intensive treatment by relevant authorities [13]. Consequently, research on kitchen garbage recycling equipment has primarily focused on the perspectives of large-scale public treatment facilities. Notable studies in this domain include that by Liu et al., who carried out a field investigation of in situ food waste treatment in canteens, markets and residential communities [14]. They argued for the necessity of a unified standard in equipment design to ensure treatment capacity. Hu et al. designed a compression treatment device with a modular structure to address the issues of high costs and space requirements associated with wet garbage treatment [15]. However, these centralised processing systems often overlook the management of food waste from household sources, which leads to community residents’ low participation rates—an indispensable factor in food waste management initiatives. Research on household or individual recycling solutions is also relatively scarce.

Therefore, the focus of this study is on household kitchen waste recycling in Chinese urban communities, characterised by limited kitchen spaces, predominantly grain-based food waste composition and a lack of established household waste recycling habits [16]. Given that waste management practices in China are still in their early stages, with traditional centralised disposal methods prevailing and household disposal not yet mainstream, it is necessary to refer to experience from other regions around the world. Therefore, this research aims to compare works existing in a wider range of regions, specifically those from a household perspective or those related to systematic solutions. These studies will be referenced and compared on the basis of their usage scenarios and treatment methods, including mechanical processing, biological processing or service systems incorporating intelligent ICT solutions.

Regarding the existing mechanical solutions for household disposal treatments, the use of under-the-sink grinders has long been regarded as a practical way to establish source reduction, which disposes the shredded waste by routing it through connected conventional sewer systems [17]. However, the use of this product has received criticism over the years because of potential adverse effects, such as increased volumes of wastewater and sludge [18]. As a response, studies have explored how such solutions can eliminate wastewater pollutants while retaining valuable materials [19]. On the other hand, research addressing food waste disposal from a biotechnological perspective is becoming more widespread. For instance, Du et al. compared seven treatment methods for kitchen food waste, arguing that biological treatment has greater advantages over non-biological treatment [20]. Zhou et al. developed a domestic composting device that shortens the typically long composting cycle by manipulating the temperature and considering the use of microbial agents [21]. The emphasis on the study of food waste bio-degradation as an alternative to conventional and less environment-friendly landfill practices is increasing. However, these solutions predominantly focus on enhancing machine performance and biotechnology, and there is a lack of research from a user experience perspective, even though users are the primary actors in household food waste management.

With the evolution of smart sensing and mobile technology, the approach to reducing food waste has expanded beyond mechanical and biological methods. ICT solutions allow for the possibility of information recording, tracking and linkage for food waste management. For example, Marques et al. proposed a multilevel internet of things (IoT)-based management system for outdoor and indoor public bins to better manage waste separation [22]. Liegeard and Manning’s research explored intelligent packaging with ink colour changing and sensors that work with intelligent appliances to remind users of the food storage conditions, thus minimising food waste [23]. Similarly, Cappelletti et al. developed an integrated smart system that guides users in their food-related daily behaviour to reduce household food waste [24]. Moreover, smart systems play a vital role in engaging stakeholders for joint management. Spyridakis et al. built a platform that connects people with surplus food to underprivileged people by using technology, volunteerism and civic engagement [25]. These research attempts imply that there are many opportunities to explore the field of integrating smart technologies into household food waste management. A comparison of the related studies is presented in Table 1.

Through comparison, potential directions for further research can be revealed. First, many studies focus narrowly on the disposal of the product itself and lack a systematic view to address the issue with relevant stakeholders or via other technological means. Second, in terms of research involving ICT solutions, many of them concentrate on utilising intelligent technologies to manage food stock while neglecting solutions for assisting residents in dealing with the already generated food waste. Lastly, there is a lack of engagement of residents as direct actors in the system, thereby limiting their motivation, participation and long-term commitment.

Given these issues, the research gap lies in allowing residents to treat household organic waste by considering user needs and creating an ICT-enabled waste management system. As such, this study takes the AHP and the TRIZ theory as guidance. The AHP is often used to examine the degree of importance of each requirement in an issue. In Balwada’s research, the AHP was used to select the best waste collection method [26]. This method is also commonly integrated with other methods, including the TRIZ theory, which systematically analyses problems to come up with innovative solutions [27,28]. Moreover, the development of wireless sensors and intelligent terminals has facilitated various smart services within urban environments [29,30], allowing for more customised requirements [31]. Leveraging the capabilities of ICT devices, this study aims to develop a smart system for urban households based on the AHP–TRIZ method. The design intends not only to focus on product efficiency in terms of weight reduction but also to provide better insights into the factors that users truly care about in the waste management process.

## 3. Research Methods

**Figure 3:**
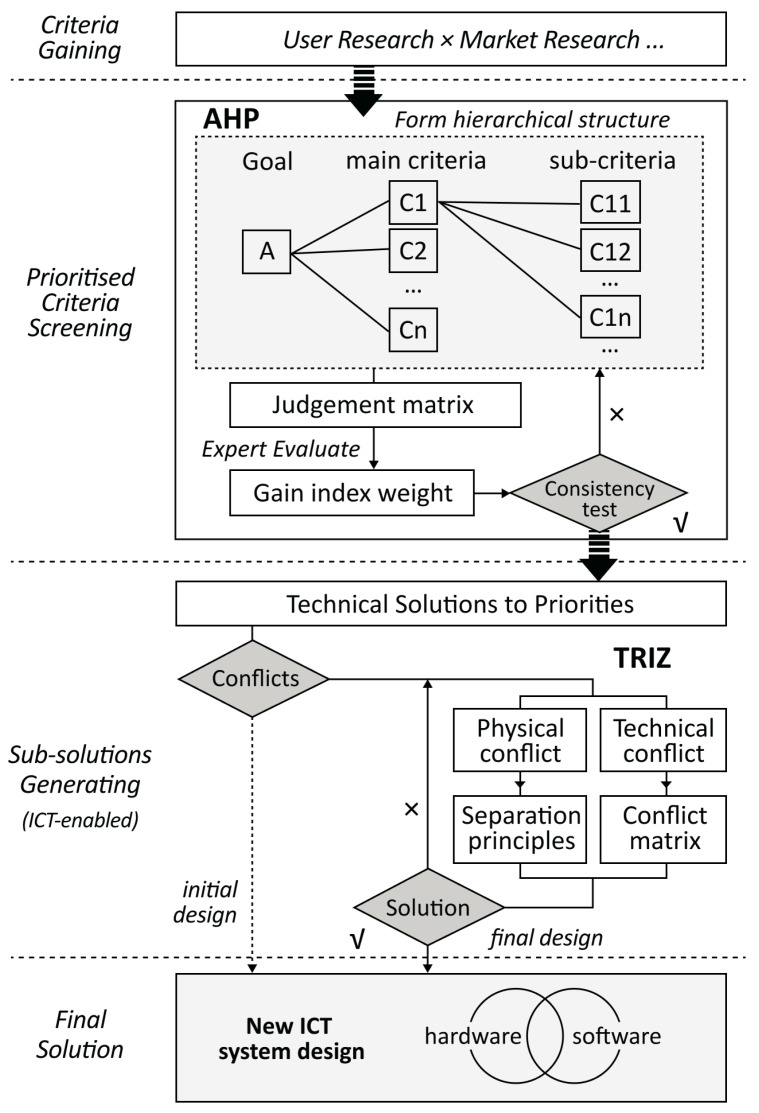
The AHP–TRIZ method model.

In the AHP–TRIZ method, the complex general goal is decomposed into multiple indexes. Each index weight is determined comparatively by following the AHP. This helps to improve the accuracy and objectivity of the identification of each weight. Then, the more vital selected indexes are transformed into specific technical requirements. The TRIZ tool is then applied to deal with the technical and physical challenges inherent in the proposition of the conceptual design. It facilitates the rapid generation of innovative concepts and helps to establish an effective thinking mode. The proposed design approach framework utilising AHP–TRIZ integration is shown in Figure 3.

### 3.1. Use AHP to Evaluate the Weight of Each Criterion

**Figure 4:**
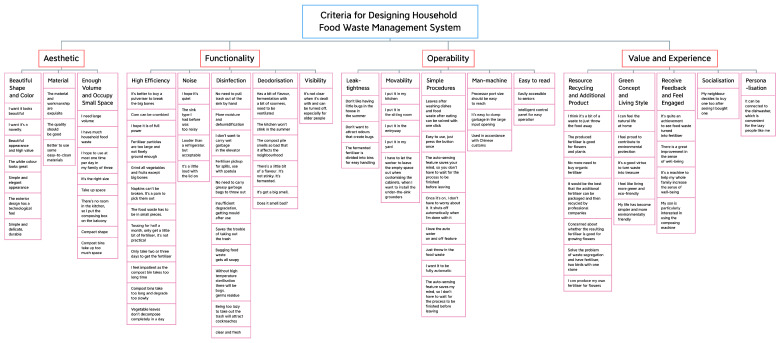
The categorised design criteria by using the affinity diagram.

**Figure 5:**
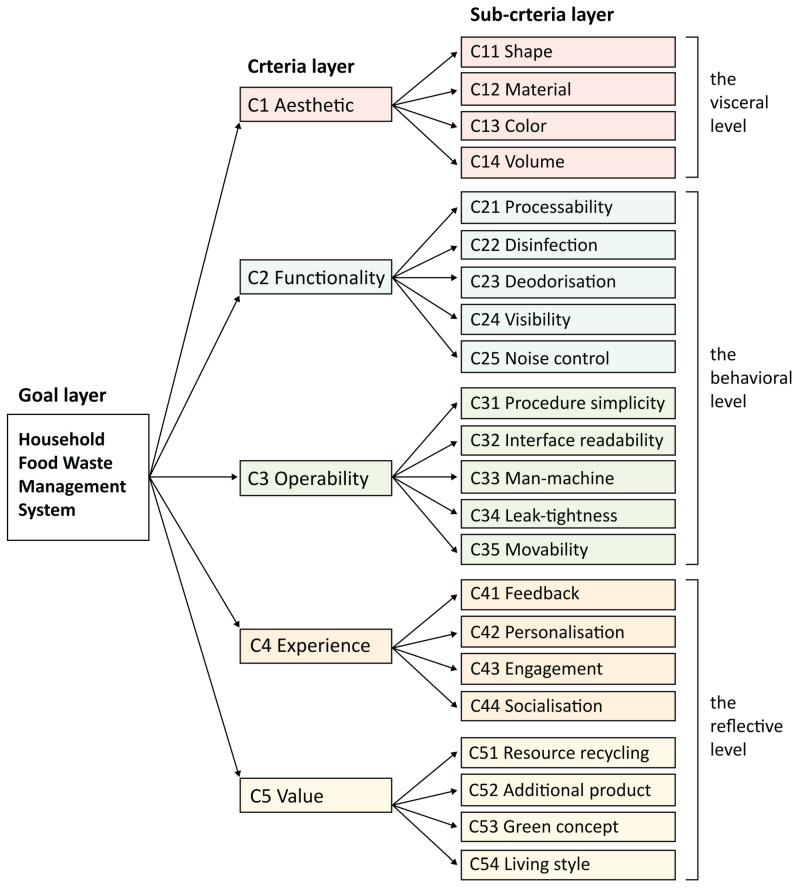
Hierarchical structure for household food waste management system.

In the first step, a round of market research of comments on 8 prevailing household food waste disposal products on Chinese e-commerce platforms (monthly sales volume of over 100) is conducted, combined with a supplement of initial interviews with 5 users who practised household food disposal, to gain the general impression and criteria of designing household food waste management system. As a result, an overall of 22 subcriteria are obtained after using the affinity diagram [32] (shown in Figure 4). Then, the subcriteria are classified into 5 main criteria according to Donald Norman’s three-level emotional design theory: the visceral, behavioural and reflective levels [33]. Similarly, Jordan defined users’ needs in terms of product characteristics, including the levels of functionality, usability and pleasure [34]. These three aspects are applicable in understanding user psychology and design requirements [35,36]:

The instinct layer focuses on users’ initial intuitive perceptions of the product, encompassing aesthetic factors, such as shape, colour, material and volume of the product. Therefore, the first main criterion for this layer is C1 Aesthetics. The behaviour layer focuses on functional utility and ease of operation, which include the interaction between the user and the product. Therefore, at the behavioural level, C2 Functionality aims at determining whether the provided functions of the system are effective and efficient, and C3 Operability refers to whether the operation process of the system is user-friendly. Moreover, the reflective layer shows the joint impact of instinct and behaviour. It is meant to establish emotional links through user interactions, as well as provide users with both psychological experience and conceptual value. Hence, another two main criteria, C4 Experience and C5 Value, are identified. The overall structure comprises 5 main criteria and 22 subcriteria, as depicted in Figure 5. The detailed explanations for each criterion can be found in Appendix A, which have also been informed to the evaluators in the subsequent scoring phase to ensure consistency.

After the requirements were clarified, the AHP questionnaire was developed, and the decision matrix was established. A total of 31 experts, including 6 design faculty, 8 master’s degree product design students, 1 food waste disposal industry salesperson and 16 experienced users, were invited to complete the questionnaire. They evaluated the importance of each indicator of the main criteria and subcriteria layers using a nine-point scale for the decision problem. The index values of the goal layer and the five main criteria form decision matrix A. In the matrix A, the index $a_{ij}$ refers to the relative importance value of indicator $a_{i}$ compared to that of indicator $a_{j}$. *n* is the number of indicators:

(1) $$A={\left(a_{ij}\right)}_{nn}=\left[\begin{array}{ccc} a_{11} & \cdots & a_{1n}\\ \vdots & \ddots & \vdots \\ a_{n1} & \cdots & a_{nn} \end{array}\right]$$

Next, 31 importance values of the comparison of each two indicators were obtained from the 31 accomplished questionnaires. Furthermore, by using the geometric average method, these 31 indexes were aggregated into $a_{ij}^{\prime }$. m is the number of experts, and $a_{ij}^{m}$ is the importance value given by each expert.

(2) $$a_{ij}^{\prime }=\sqrt[{31}]{\prod_{m=1}^{31}a_{ij}^{m}}$$

A new aggregated judgment matrix A’ was then formed. The matrix $A_{C1-C5}^{\prime }$ is an example. The calculated indexes are shown in Table 2, and the calculated indexes of other subcriteria’s judgement matrices are shown in Appendix B.

Then, the geometric mean of each criterion $V_{i}$ and weight vector *W* was obtained using the geometric average calculating method:

(3) $$V_{i}=\sqrt[{n}]{\prod_{j=1}^{n}a_{ij}^{\prime }},\left(i=1,2,3,4,5\right)$$

(4) $$w_{i}=\frac{V_{i}}{\sum_{i=1}^{n}V_{i}},W=\left(w_{1},w_{2},\cdots ,w_{n}\right)$$

To ensure the judgments made in all the matrices are reasonable, the consistency test follows the next step. Consistency ratios (CR) were identified for each of the matrices calculated using the largest eigenvalue $\lambda_{max}$ and the corresponding eigenvector $w_{i}$.

(5) $$\lambda_{max}=\frac{1}{n}\cdot \sum_{i=1}^{n}\frac{{\left(AW\right)}_{i}}{w_{i}}$$

(6) $$CI\left(ConsistencyIndex\right)=\frac{\lambda_{max}-n}{n-1}$$

(7) $$CR\left(ConsistencyRatio\right)=\frac{CI}{RI}$$

The calculated CRs of each criterion were less than 0.1 (see Table 3), which means that the importance evaluation results of each index are reasonable.

**Figure 6:**
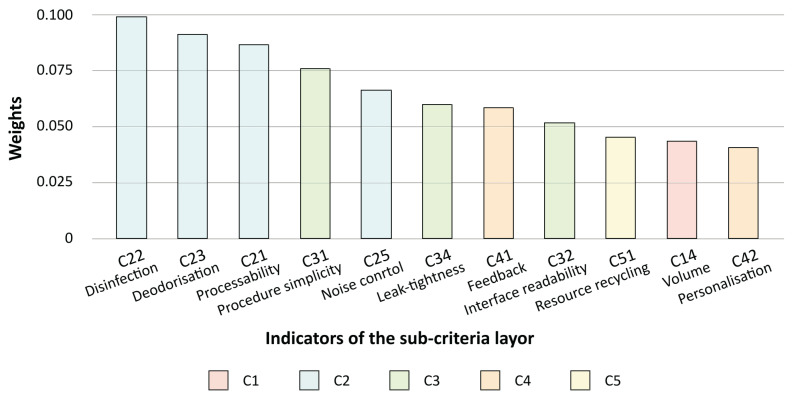
Weights of the first half of the indicators.

Similar calculations of other matrices were conducted in the same way. The weights and rankings of each criterion are shown in Table 4:

The weights of C2 Functionality and C3 Operability were significantly higher than those of the other requirements in the main criterion layer, so these two factors should be given more attention. For the subcriteria, the first half were selected as key issues for the design of the system; see Figure 6.

In the analysis of conventional technical solutions for the first 11 criteria, some conflicts can be identified. For instance, the product needs to incorporate modules with multiple functionalities while keeping the overall volume as compact as possible. Therefore, the TRIZ tool is introduced to address the conflicts existing among these criteria. Moreover, considering the objective of developing a smart system in this paper, there is a tendency to prioritise ICT-related solutions that align with TRIZ principles.

### 3.2. Use TRIZ to Solve Conflicts

There are two main types of conflicts: physical conflicts, which are addressed through four separation principles, and technical conflicts, which are solved through the invention principle and the conflict matrix composed of 39 technical parameters. Under the proposed problem of this study, two sets of physical conflicts and two sets of technical conflicts were identified and transformed into the TRIZ problem model, as shown in Table 5:

#### 3.2.1. *Physical Conflict 1*

The addition of catalytic fungi is commonly used to accelerate food waste decomposition [37]. However, the decomposition process also leads to the rapid proliferation of harmful bacteria. A sufficient amount of catalytic fungi and as little harmful bacteria as possible should be present. This requirement of simultaneously increasing and decreasing bacterial populations creates an evident conflict.

According to the separation upon condition principle, these two types of microorganisms can be distinguished based on their differences in temperature tolerance parameters. Harmful bacteria belong to the group of thermolabile bacteria, while decomposing microorganisms belong to the group of thermophilic bacteria [38]. Therefore, a potential solution in which a temperature-sensitive heating control system is installed in the decomposition zone is proposed. This system consists of a temperature sensor and electric heating tubes uniformly arranged on the inner walls of the chamber. It continuously and intermittently heats the internal content to over 60°C during the decomposition process, thus ensuring that harmful bacteria can be effectively killed but that the catalytic fungi remain active.

#### 3.2.2. *Physical Conflict 2*

There is a conflict between the requirements of deodorisation and leak tightness. On the one hand, deodorisation requires odours inside to be eliminated through ventilation. On the other hand, leak tightness requires the product to have good sealing properties in order to prevent internal odours and waste liquids from leaking into the external environment. This inconsistency leads to a physical conflict.

According to the separation in space principle, different modules can be set up to optimise the product’s sealing structure, including a sealed chamber and an air circulation system for deodorisation and dehumidification. The air circulation system can control the flow and direction of incoming and outgoing air. Inside, an ozone disinfection device is added to oxidise and decompose the odour gas, and a high-efficiency particulate air filter is used to prevent particles in the gas from leaking out. This design prevents the diffusion of odours by filtering and treating the gas while maintaining good sealing.

#### 3.2.3. *Technical Conflict 1*

The goal is to create a complex system that addresses multiple needs of noise reduction, dehumidification, disinfection, sterilisation and storage. This results in an increase in the product’s volume and occupied space, which is not ideal for the limited space typically found in Chinese urban kitchens.

According to segmentation principle no. 1, the product can be divided into several modules based on main product functions. The modular design allows users to temporarily disassemble modules that are not currently needed. For example, they can choose whether to install a storage module based on their own requirements, effectively reducing the overall volume and space occupied by the product. This provides a more flexible and adaptable solution for Chinese households with space limitations in their kitchens.

#### 3.2.4. *Technical Conflict 2*

The conventional manual recycling process usually includes multiple trivial steps, such as collecting, dumping, adding catalysts, regular turning and stirring, waiting and taking out. These laborious procedures need to be simplified. However, such simplification keeps users who have different needs or who enjoy the composting experience from setting parameters according to their personal habits.

Using preliminary principle no. 10, the product supports several preset options through a mobile application. Sensors and clips collect information about the recycling process, which is then transferred and displayed on the user’s mobile app, merging and automating complex procedures for user convenience. Users can choose their preferred presets and select between the Quick Mode for rapid waste reduction or the Standard Mode for thorough decomposition. Based on this, the data recorded by sensors, including equipment status, waste types and disposal capacity, together with user inputs in the mobile app, are to be analysed and learned. By utilising deep learning techniques [39], the system can recognise patterns and trends in the household’s dietary habits and waste disposal practices, thus automatically adjusting working modes and offering personalised recommendations for food consumption and disposal.

## 4. Design of the Household Food Waste Management Smart System

**Figure 7:**
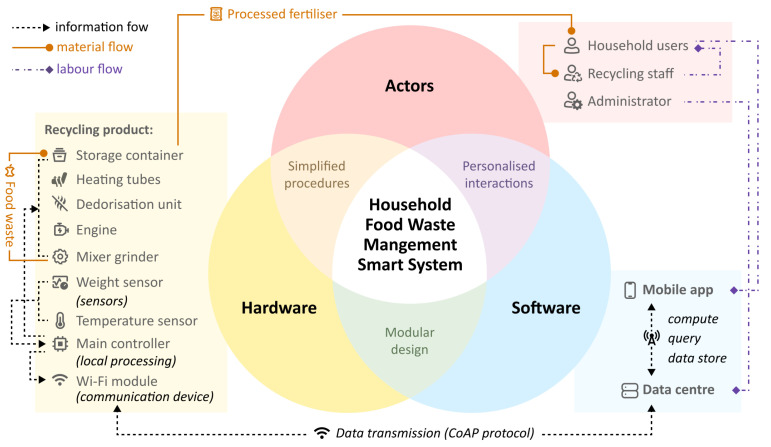
Proposed household food waste management system map.

Based on the AHP–TRIZ model, a new food waste management system that uses intelligent techniques is illustrated in Figure 7, including a data centre, a semi-automated recycling product and a mobile application. Briefly, the hardware recycling product is responsible for fulfilling user requirements regarding hygiene and waste disposal efficiency. On the other hand, the software component, including the mobile app, the sensors that collect disposal data and the cloud centre that processes data, utilises machine learning techniques to address user demands for simplified operation and personalised settings.

**Figure 8:**
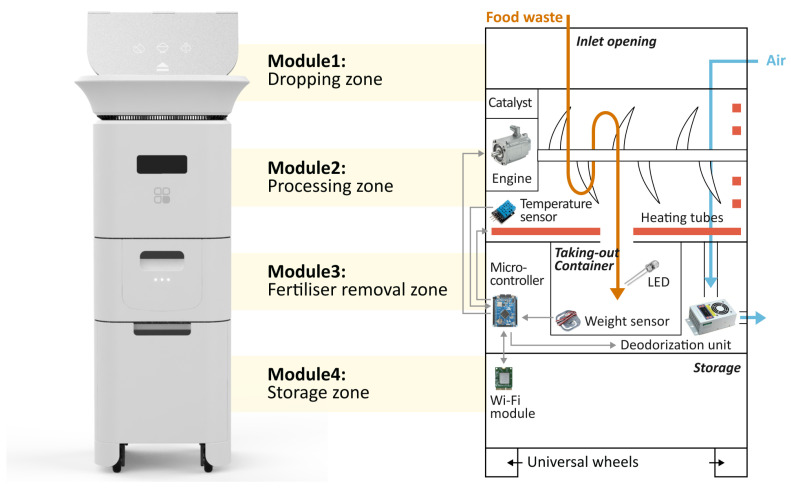
Structure of the recycling product.

**Figure 9:**
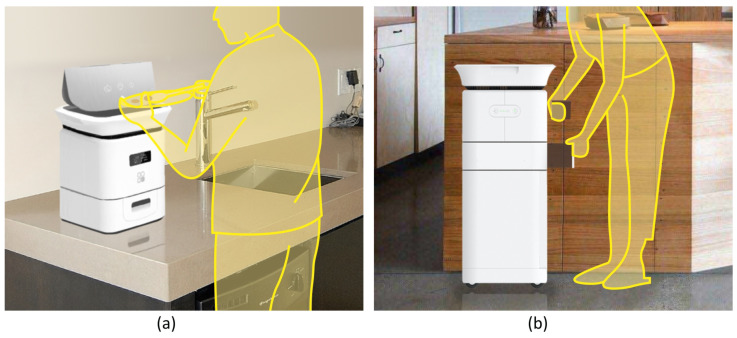
Usage in a household environment. (**a**) Put on the countertop. (**b**) Flexible and movable.

**Figure 10:**
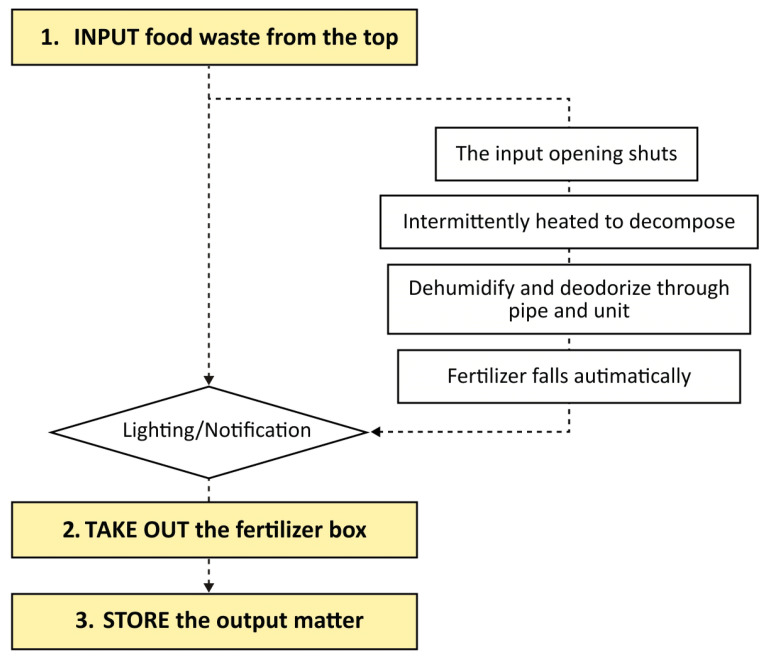
Steps for usage.

**Figure 11:**
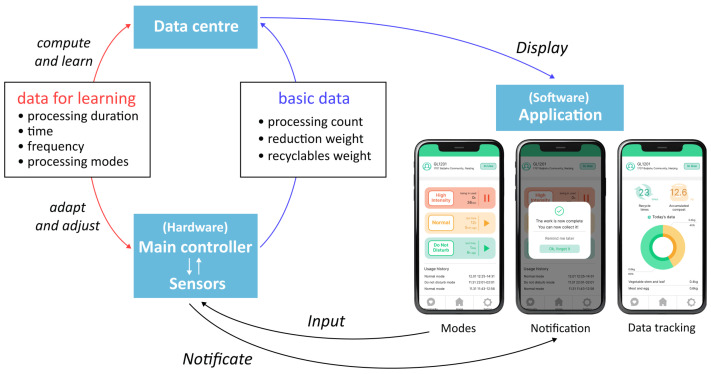
The software component in the system.

The highlights of recycling management system innovation are as follows:

(1) *Modular Design:* As illustrated in Figure 8, the recycling product contains four modules arranged from top to bottom: the dropping zone, the processing zone, the fertiliser removal zone and the storage zone, enabling multiple key subfunctions. The dropping zone is designed for user input convenience with an expandable large opening and stainless steel material to prevent stains from liquid leakage. The processing zone contains stirring and grinding blades, a small container for microbial catalysts and an embedded temperature and humidity sensor. The main controller regulates heating pipes for the sterilisation of harmful bacteria and for maintaining the activity of decomposition microorganisms. The fertiliser removal zone stores processed products and has a weight sensor that sends capacity reminders via a Wi-Fi module. The bottom storage zone stores packaged value-added products, as well as tools such as gloves, compost packages and small shovels. It is also equipped with universal wheels for movability. The modular design of the recycling product helps to reduce the space it occupies. In Figure 9, when the storage zone is removed, the product can be placed on the countertop. By attaching universal wheels at the bottom, the product can be easily moved around the kitchen, balcony or other areas. This flexibility caters to the specific environment of Chinese households.
(2) *Simplified User Workflow:* Designed from a user-centric perspective, this system aims to facilitate long-term user engagement by minimising usage complexity and costs. Unlike traditional home composting processes in which users are required to regularly monitor, turn and control catalysts, this system incorporates sensors and automated mechanisms to simplify the intermediate steps. As depicted in Figure 10, the process can be summarised as input–take out–store. Users begin by inputting their food waste into the recycling product. The main controller operates the stirring and grinding device, while the temperature sensor and heat pipes regulate microbial activity and decomposition efficiency. Additionally, an air circulation system connected to an odour and moisture removal device automatically maintains hygiene and cleanliness across different modules of the machine. When the value-added product fills up the container, the heavy sensor provides feedback to the main controller, triggering a lighting indicator on the machine and sending a notification to the user’s mobile app. At this stage, users only need to take out the processed fertiliser and package it for storage without any check halfway through the process, awaiting scheduled collection by the recycling staff.
(3) *Personalised User Interaction:* On the other hand, simplifying the operational steps does not mean standardising user interactions during the food waste recycling process. User engagement in the recycling process can be personalised and enriched through the functions provided by the mobile application as well as adaptive product processing.
  As illustrated in Figure 11, the data recognised and collected by the sensors in the hardware component can be broadly categorised into two types: basic data that can be presented to users and data analysed by the system to better understand user habits. Considering that household waste often contains sensitive personal information, data transmission is conducted using the CoAP security protocol specifically designed for IoT applications [22,40]. On the one hand, basic data, including processing count, reduction weight and recyclables weight, are made accessible to users through their mobile apps, and visually displayed. This allows users to conveniently track their disposal progress and history, imperceptibly fostering a sense of accomplishment and environmental consciousness. Additionally, users have the flexibility to choose between Quick Mode and Standard Mode, along with a do-not-disturb function, via the presetting feature, catering to their individual needs. On the other hand, data related to processing duration, time, frequency, corresponding processing modes and user input in the mobile app are recognised and processed using deep learning techniques [41,42]. This empowers the system to analyse and learn different users’ waste disposal habits, enabling it to adaptively adjust different households’ waste processing modes. As a result, users can benefit from automated and customised kitchen waste disposal modes tailored to their respective food consumption habits.

Compared to traditional composting methods, the new design offers significant advantages. In processing 1 kg of kitchen waste (800 g of fruit and vegetable peels, 150 g of grain and 50 g of meat residues), 175 g of fertiliser is produced within 10 h, achieving a higher reduction rate of 82% and a faster process than the traditional methods. For instance, in a month, a family of three can reduce approximately 37 kg of waste into around 6.5 kg of usable fertiliser. Furthermore, follow-up interviews with initial participants revealed a notable increase in willingness to use the new system, especially with high expectations for the ICT-related features, like tracking disposal status and adaptively streamlining processing procedures. The result demonstrates that our optimised system offers an effective and efficient solution for food waste management and has the potential for broader applications in promoting sustainable and eco-friendly living.

## 5. Discussion

### 5.1. A User-Centric Perspective to Promote Sustained Engagement

The implication of the term “user-centric” in this study is twofold. Firstly, it involves addressing users’ genuine needs, which differs from conventional approaches that solely focus on mechanical or biological ways to accelerate food waste decomposition. For instance, from the AHP analysis in Section 3.1, this study recognises that users prioritise the hygiene of the recycling product as their first concern over mechanical improvements for faster decomposition. This is because, unlike centralised recycling systems, the target location of the system in this study is within households, directly impacting users’ personal living environments and making hygiene their foremost consideration. By contrast, centralised recycling facilities are typically located in public spaces, leading residents to prioritise waste disposal convenience, while government institutions tend to emphasise processing efficiency. Consequently, solutions aimed at ensuring sanitation will be developed with more targeted aims. In our design, the corresponding solutions include periodic sterilisation through heating, an air circulation system equipped with a deodorisation unit and a larger input opening to facilitate user convenience. By understanding and catering to these real user needs, users become more engaged and motivated as the first practitioners to conduct household food waste management.

Secondly, the term "user-centric" means enhancing users’ operational capability. This can be achieved by reducing the difficulty regarding user interaction and incorporating personalised features, such as offering preset modes and personalised adaptive tracking mechanisms. These solutions address the issue of users’ unfamiliarity with household food waste recycling operations, reducing barriers that hinder their engagement in recycling, therefore fostering long-term habits and sustaining their recycling behaviour.

Adopting a user-centric perspective that addresses genuine user needs and enhances operational capability is vital in designing a food waste recycling system. By acknowledging users as active practitioners and ensuring that the design aligns with their authentic requirements, sustained engagement and effective recycling behaviour can be fostered.

### 5.2. ICT-Enabled Solutions to Balance Conflicts within the System

Integrating smart devices, such as sensors and mobile terminals, opens up innovative possibilities for addressing complex and conflicting requirements of household management issues. New approaches to problem solving can be explored by leveraging ICT-enabled solutions.

For example, physical conflict 1 can be resolved by utilising temperature sensors to detect changes in the status of decomposed matter. By controlling the heating tubes based on these readings, a balance between two microbial populations can be achieved, ensuring efficient decomposition. Moreover, in technical conflict 2, automation and sensor-based monitoring can simplify user interactions. By incorporating predefined control patterns and sensors, users can effortlessly select different recycling modes without complex manual adjustments. Furthermore, the system can gradually adapt to user operations by intelligently learning their usage habits, thereby directly providing disposal solutions that align with their operational preferences and food consumption.

These ICT-enabled solutions not only streamline users’ management processes and improve their willingness to conduct recycling but also expand the capability of TRIZ principles in addressing conflicts within a system. These solutions offer efficient ways to balance conflicting subcriteria’s solutions, resulting in optimised waste management processes and improved user experiences.

### 5.3. Hardware–Software Integration to Shape the New Management System

In the context of smart management, product design and manufacturing can no longer be the only sources of competitive advantage and differentiation. Integrating sensing systems and mobile service technologies offers a broader perspective for problem solving.

Traditional solutions for food waste management typically involve under-the-sink grinders or compostors, focusing on improving operational performance and efficiency from mechanical and biological perspectives. However, there has been limited exploration of utilising intelligent technologies to assist users in smooth and effortless household food waste recycling, aiming for a more comfortable and enjoyable experience. The proposed solution in this paper aims to generate a smart management system by incorporating hardware and software, guided by user-centric criteria obtained through the AHP. Through sensors embedded in the hardware, data such as the timing, frequency and disposal modes can be collected, enabling the acquisition of user behaviour patterns and preferences. User input preferences, such as specific processing methods, can be recorded through the software. By training and pattern recognition using extensive user data, the system can gradually learn and understand individualised preferences and disposal habits, thereby achieving a certain level of automation and providing personalised services to users.

By embracing a system-level perspective and leveraging the advantages of hardware-software integration, the food waste management system becomes more than just a mechanical solution. It facilitates efficiency, enhanced user experience, data-driven insights and seamless integration within the broader ecosystem of smart living.

## 6. Conclusions

Utilising food waste resources is a crucial step towards green development and green living. Apart from the commonly adopted centralised waste management mode, exploring how household-level food waste recycling can be facilitated is essential as the home is the most direct and decentralised source of food waste.

This study uses the AHP–TRIZ method to design an intelligent household food waste management system from a user-centric perspective. The AHP helps to identify genuine user needs and objectively prioritises requirements by calculating criterion weights. From the analysis, we found that users primarily focus on fundamental aspects, such as functionality and operability, when it comes to household recycling. Demands related to experience and value may emerge once the foundational aspects of functionality and operability are well established. Additionally, TRIZ tools are employed to address the physical and technical conflicts that arise from common solutions to these requirements. TRIZ principles have been applied and expanded in the construction of the system. Through the integration of ICT techniques, more solutions that have not widely been adopted in the field of food waste processing are generated.

The outcome is a smart food waste management system comprising a recycling product for food waste reduction, a mobile application for user engagement, and a data centre for data transmission, computing, and storage. This system effectively enhances the recycling rate of household food waste while promoting interaction between individuals and intelligent appliances.

In future research, the limitations of the current study need to be addressed and improved in two aspects. Firstly, at the method level of the system development, it is necessary to consider incorporating ICT solutions to further innovate and advance the current TRIZ part. The current TRIZ principles would break away from the predominantly mechanical development approach and facilitate a more direct reference for the development of other intelligent systems. Secondly, at the solution level, there is a need to optimise the current preliminary solutions in terms of how artificial intelligence can learn user processing disposal patterns and provide reasonable recommendations. Additionally, conducting a wider range of user testing is essential to further validate the effectiveness of the proposed solution, thus making this research a pilot for developing other household intelligent management systems.

## Figures and Tables

**Table 1:** Comparison between related works.

Work	Scenarios	Treatment	ICT	Goal
Bernstad et al. [17]	Household: under the sink	Mechanical: grind and discharge into sewers	-	• reduce waste • create methane
Cecchi and Cavinato [19]	Public: under the sink to waste stations	Mechanical and biological: grind and then dispose in treatment plant	-	• avoid transportation • energy recovery
Zhou et al. [21]	Household: kitchen composting bins	Biological: high- temperature composting	-	• food waste reduction • make value-added products
Marques et al. [22]	Public: outdoor and indoor bins	ICT-related: sensing and recognition	RFID sensors, cloud platform, etc.	• correct separation • a simultaneous bin network
Liegeard and Manning [23]	Household: kitchen smart fridges	ICT-related: packaging for food track	Biosensors, RFID, a control unit, etc.	• manage stock control • reduce food waste
Cappelletti et al. [24]	Household: kitchen smart fridges	ICT-related: food stock track	A smart fridge an application	• food waste reduction • healthy diet
Spyridakis et al. [25]	Public: campus dining halls	ICT-related: pick-up and delivery	An open-source website	• sharing concept • reduce food waste

**Table 2:** Pairwise comparison of criteria in matrix $A_{C1-C5}^{\prime }$.

	C1	C2	C3	C4	C5
**C1**	1.00	0.44	0.42	0.68	1.06
**C2**	2.29	1.00	1.89	3.28	2.74
**C3**	2.38	0.53	1.00	2.69	1.34
**C4**	1.48	0.30	0.37	1.00	1.71
**C5**	0.95	0.36	0.75	0.58	1.00

**Table 3:** Results of the consistency ratios.

	A	C1	C2	C3	C4	C5
CI	0.040	0.004	0.012	0.036	0.034	0.023
RI	1.12	0.89	1.12	1.12	0.89	0.89
CR	0.036	0.005	0.011	0.032	0.038	0.026

**Table 4:** Results of comprehensive weights.

Criteria	Sub-Criteria	Weight	Ranking
C1: Aesthetic 0.120	C11: Shape	0.031	14
	C12: Material	0.031	15
	C13: Colour	0.015	21
	C14: Volume	0.043	10
C2: Functionality 0.374	C21: Processability	0.087	3
	C22: Disinfection	0.099	1
	C23: Deodorisation	0.091	2
	C24: Visibility	0.031	16
	C25: Noise control	0.066	5
C3: Operability 0.243	C31: Procedure simplicity	0.076	4
	C32: Interface readability	0.051	8
	C33: Man–machine	0.038	12
	C34: Leak-tightness	0.060	6
	C35: Movability	0.019	19
C4: Experience 0.140	C41: Feedback	0.058	7
	C42: Personalisation	0.041	11
	C43: Engagement	0.028	17
	C44: Socialisation	0.014	22
C5: Value 0.123	C51: Resource recycling	0.045	9
	C52: Additional product	0.019	20
	C53: Green concept	0.024	18
	C54: Living style	0.035	13

**Table 5:** TRIZ problem transformation of the conflicts.

No.	Type	Paradoxical Attributes	General Engineering Parameters	TRIZ Principles
1	Physical conflict	Disinfection–no germ	31 Harmful side effects	Separation upon condition
		Processability–contain germ		
2	Physical conflict	Deodorisation–let in air	32 Manufacturability	Separation in space
		Leak-tightness–air-proof		
3	Technical conflict	Requirement of multi-function	36 Complexity of device	no. 1 Segmentation
		Volume	8 Volume of non-moving object	
4	Technical conflict	Procedure simplicity	33 Convenience of use	no. 10 Preliminary
		Personalisation	24 Loss of information	

## Data Availability

The data used to support the findings of this study are available from the corresponding author upon request.

## Appendix A

**Table A1:** Explanation for AHP criteria.

No.	Criterion	Explanation
**C1**	**Aesthetic**	The visual appeal of the products within the system.
C11	Shape	Physical form (round or square; curvaceous or rectilinear).
C12	Material	Choice of construction materials.
C13	Colour	Use of colours in the system’s design.
C14	Volume	Capacity or size of the product.
**C2**	**Functionality**	The system’s ability to perform its intended tasks.
C21	Processability	Efficient handling and processing of food waste.
C22	Disinfection	Ensuring hygienic treatment of waste.
C23	Deodorisation	Elimination of unpleasant odours.
C24	Visibility	Clear visibility of the waste management process.
C25	Noise control	Minimisation of noise generated during operation.
**C3**	**Operability**	The ease of use and operation of the system.
C31	Procedure simplicity	The simplicity of system operation procedures.
C32	Interface readability	Clear and user-friendly interface design.
C33	Man–machine	The scale of the product lines with human operations.
C34	Leak-tightness	Prevention of any leakage or spillage.
C35	Movability	Easy movement or portability of the product.
**C4**	**Experience**	User’s overall experience when operating the system.
C41	Feedback	Providing feedback to users (reminder, notification, etc.)
C42	Personalisation	Customisation options for individual preferences.
C43	Engagement	Encouraging user involvement and participation.
C44	Socialisation	Promoting social interactions and community engagement.
**C5**	**Value**	The system’s social impact and conceptual value.
C51	Resource recycling	Substantial reduction in food waste.
C52	Additional product	Creation of additional valuable products from food waste.
C53	Green concept	Raising users’ environmentally friendly awareness.
C54	Living Style	Changing the way users live.

## Appendix B

**Table A2:** Pairwise comparison of criteria in matrices C1′–C5′.

matrix $C1^{\prime }$	C11	C12	C13	C14	
**C11**	1.00	1.11	1.94	0.71	
**C12**	0.90	1.00	2.42	0.67	
**C13**	0.52	0.41	1.00	0.38	
**C14**	1.42	1.49	2.62	1.00	
**matrix $C2^{\prime }$**	**C21**	**C22**	**C23**	**C24**	**C25**
**C21**	1.00	1.05	0.84	2.21	1.63
**C22**	0.95	1.00	1.38	3.31	1.41
**C23**	1.19	0.73	1.00	3.34	1.35
**C24**	0.45	0.30	0.30	1.00	0.41
**C25**	0.61	0.71	0.74	2.44	1.00
**matrix $C3^{\prime }$**	**C31**	**C32**	**C33**	**C34**	**C35**
**C31**	1.00	1.50	1.99	1.29	3.81
**C32**	0.67	1.00	1.62	0.75	2.46
**C33**	0.50	0.62	1.00	0.67	2.17
**C34**	0.78	1.33	1.48	1.00	3.02
**C35**	0.26	0.41	0.46	0.33	1.00
**matrix $C4^{\prime }$**	**C41**	**C42**	**C43**	**C44**	
**C41**	1.00	2.18	1.81	3.10	
**C42**	0.46	1.00	1.94	3.40	
**C43**	0.55	0.52	1.00	2.20	
**C44**	0.32	0.29	0.45	1.00	
**C51**	1.00	2.61	2.74	0.92	
**C52**	0.38	1.00	0.75	0.60	
**C53**	0.40	1.34	1.00	0.88	
**C54**	1.08	1.66	1.14	1.00	
